# Supplementary material for: Followers Are Not Enough: A Multifaceted Approach to Community Detection in Online Social Networks
Source: PLoS One. 2015 Aug 12;10(8):e0134860. doi: 10.1371/journal.pone.0134860 (PMC4534395; doi:10.1371/journal.pone.0134860)
Supplement: S1 Text — (PDF) [file pone.0134860.s001.pdf]

Text S1 – Supporting Information for the Paper "Followers Are Not Enough: A Multifaceted Approach to Community Detection in Online Social Networks" by D. Darmon, E. Omodei, J. Garland

1 Transfer entropy and its estimation from data.

Let $\{X_t\}$ and $\{Y_t\}$ be two strong-sense stationary stochastic processes. Recall that a stochastic process is strong-sense stationary if the joint distribution for the process evaluated at finitely many time points is invariant to an overall timeshift [3]. In our work, these would correspond to the activities, $X_t(u)$ and $X_t(v)$, of two users u and v. We use the notation X_{t-k}^{t} to denote the values of the stochastic process from time $t-k$ to time t, $X_{t-k}^{t} = (X_{t-k}, X_{t-(k-1)}, \ldots, X_{t-1}, X_t)$. The lag-$k$ transfer entropy [6] of Y on X is defined as

$$\mathrm{TE}_{Y \to X}^{(k)} = H\left[X_t | X_{t-k}^{t-1}\right] - H\left[X_t | X_{t-k}^{t-1}, Y_{t-k}^{t-1}\right], \tag{1}$$

where

$$H\left[X_t | X_{t-k}^{t-1}\right] = -E\left[\log_2 p(X_t | X_{t-k}^{t-1})\right] \tag{2}$$

and

$$H\left[X_t | X_{t-k}^{t-1}, Y_{t-k}^{t-1}\right] = -E\left[\log_2 p(X_t | X_{t-k}^{t-1}, Y_{t-k}^{t-1})\right] \tag{3}$$

are the usual conditional entropies over the conditional (predictive) distributions $p(x_t | x_{t-k}^{t-1})$ and $p(x_t | x_{t-k}^{t-1}, y_{t-k}^{t-1})$. This formulation was originally developed in [6], where transfer entropy was proposed as an information theoretic measure of *directed* information flow. Formally, recalling that $H\left[X_t | X_{t-k}^{t-1}\right]$ is the uncertainty in X_t given its values at the previous k time points, and that $H\left[X_t | X_{t-k}^{t-1}, Y_{t-k}^{t-1}\right]$ is the uncertainty in X_t given the joint process $\{(X_t, Y_t)\}$ at the previous k time points, transfer entropy measures the reduction in uncertainty of X_t by including information about Y_{t-k}^{t-1}, controlling for the information in X_{t-k}^{t-1}. By the 'conditioning reduces entropy' result [1]

$$H[X|Y, Z] \leq H[X|Y], \tag{4}$$

we can see that transfer entropy is always non-negative, and is zero precisely when

$$H\left[X_t | X_{t-k}^{t-1}\right] = H\left[X_t | X_{t-k}^{t-1}, Y_{t-k}^{t-1}\right],$$

in which case knowing the past k lags of Y_t does not reduce the uncertainty in X_t. If the transfer entropy is positive, then $\{Y_t\}$ is considered causal for $\{X_t\}$ in the Granger sense [2].

When estimating transfer entropy from finite data, we will assume that the process $\{(X_t, Y_t)\}$ is jointly stationary, which gives us that

$$p(x_t | x_{t-k}^{t-1}) = p(x_{k+1} | x_1^k) \tag{5}$$

and

$$p(x_t|x_{t-k}^{t-1}, y_{t-k}^{t-1}) = p(x_{k+1}|x_1^k, y_1^k) \tag{6}$$

for all t. That is, the predictive distribution only depends on the past, not on when the past is observed. Given this assumption, we compute estimators for $p(x_{k+1}|x_1^k)$ and $p(x_{k+1}|x_1^k, y_1^k)$ by 'counting': for each possible marginal and joint past x_1^k and (x_1^k, y_1^k), we count the number of times a future of type x_{k+1} occurs, and normalize to obtain the appropriate estimators of the one-step-ahead predictive distributions. Call these estimators $\hat{p}(x_{k+1}|x_1^k)$ and $\hat{p}(x_{k+1}|x_1^k, y_1^k)$. Then the plug-in estimator for the transfer entropy is

$$\widehat{\mathrm{TE}}_{Y\to X}^{(k)} = \hat{H}\left[X_t|X_{t-k}^{t-1}\right] - \hat{H}\left[X_t|X_{t-k}^{t-1}, Y_{t-k}^{t-1}\right] \tag{7}$$

where we use the plug-in estimators $\hat{H}\left[X_t|X_{t-k}^{t-1}\right]$ and $\hat{H}\left[X_t|X_{t-k}^{t-1}, Y_{t-k}^{t-1}\right]$ for the entropies. It is well known that the plug-in estimator for entropy is biased [5]. To account for this bias, we use the Miller-Madow adjustment to the plug-in estimator [4]. For a random variable X taking on finitely many values from an alphabet $\mathcal{X}$, the Miller-Madow estimator is

$$\tilde{H}[X] = \hat{H}[X] + \frac{|\hat{\mathcal{X}}| - 1}{2n} \tag{8}$$

where $|\hat{\mathcal{X}}|$ is the number of observed symbols from the alphabet $\mathcal{X}$ and n was the number of samples used to estimate $\hat{H}[X]$. The definition of transfer entropy (1) can be rewritten in terms of joint entropies as

$$\begin{aligned} TE_{Y\to X}^{(k)} &= H[X_t|X_{t-k}^{t-1}] - H[X_t|X_{t-k}^{t-1}, Y_{t-k}^{t-1}] && (9) \\ &= H[X_t, X_{t-k}^{t-1}] - H[X_{t-k}^{t-1}] - H[X_t, X_{t-k}^{t-1}, Y_{t-k}^{t-1}] + H[X_{t-k}^{t-1}, Y_{t-k}^{t-1}], && (10) \end{aligned}$$

We then apply the Miller-Madow adjustment individually to each of the entropy terms. For example, for the first term, we have

$$\tilde{H}[X_t, X_{t-k}^{t-1}] = \tilde{H}[X_{t-k}^{t}] = \hat{H}[X_{t-k}^{t}] + \frac{|\widehat{\mathcal{X}^{k+1}}| - 1}{2n}, \tag{11}$$

where $|\widehat{\mathcal{X}^{k+1}}|$ is the number of $(k+1)$-tuples we actually observe (of the 2^{k+1} possible tuples). Doing this for each term, the overall Miller-Madow estimator for the transfer entropy is

$$\begin{aligned} \widetilde{TE}_{Y\to X}^{(k)} &= \tilde{H}[X_t|X_{t-k}^{t-1}] - \tilde{H}[X_t|X_{t-k}^{t-1}, Y_{t-k}^{t-1}] && (12) \\ &= \tilde{H}[X_t, X_{t-k}^{t-1}] - \tilde{H}[X_{t-k}^{t-1}] - \tilde{H}[X_t, X_{t-k}^{t-1}, Y_{t-k}^{t-1}] + \tilde{H}[X_{t-k}^{t-1}, Y_{t-k}^{t-1}]. && (13) \end{aligned}$$

One possible problem with this estimator is that it can result in *negative* estimates of entropies. That usually occurs when $\hat{H}$ is very small. In these cases, we set the estimator to zero.

References

[1] Thomas M Cover and Joy A Thomas. *Elements of information theory*. Wiley-Interscience, 2006.

[2] Clive William John Granger. Economic processes involving feedback. *Inform. Control*, 6(1):28–48, 1963.

[3] Geoffrey Grimmett and David Stirzaker. *Probability and random processes*. Oxford University Press, 2001.

[4] George A Miller. Note on the bias of information estimates. In H Quastler, editor, *Information theory in psychology II-B*, pages 95–100. Glencoe, Illinois: Free Press, 1955.

[5] Liam Paninski. Estimation of entropy and mutual information. *Neural Comput.*, 15(6):1191–1253, 2003.

[6] Thomas Schreiber. Measuring information transfer. *Phys. Rev. Lett.*, 85(2):461–464, 2000.
